# Expression of Concern: Human and Environmental Dangers Posed by Ongoing Global Tropospheric Aerosolized Particulates for Weather Modification

**DOI:** 10.3389/fpubh.2016.00155

**Published:** 2016-07-19

**Authors:** Frontiers Editorial Office

With this notice, Frontiers states its awareness of several complaints and serious allegations surrounding the article, “Human and Environmental Dangers Posed by Ongoing Global Tropospheric Aerosolized Particulates for Weather Modification,” published on 30 June, 2016. Our Chief Editors, Joav Merrick and Anwar Huq, will direct an investigation in full accordance with our complaints procedures. The situation will be updated as soon as the investigation is complete.

